# Spontaneous Hemoperitoneum From a Ruptured Gastrointestinal Stromal Tumor

**DOI:** 10.7759/cureus.9338

**Published:** 2020-07-22

**Authors:** Jordan Shively, Charles Ebersbacher, William T Walsh, Matthew T Allemang

**Affiliations:** 1 General Surgery, Cleveland Clinic South Pointe Hospital, Warrensville Heights, USA; 2 General Surgery, Ohio University Heritage College of Osteopathic Medicine, Warrensville Heights, USA

**Keywords:** gastrointestinal stromal tumor, spontaneous hemoperitoneum, gist, gastrointestinal stromal tumor (gist)

## Abstract

This is a case report of a ruptured gastrointestinal stromal tumor (GIST) presenting as spontaneous hemoperitoneum. The patient was a 63-year-old female with a past medical history of hypertension and ulcerative colitis who presented to the emergency department with worsening epigastric pain. The patient denied history of trauma, previous surgeries, or forceful vomiting. She was not on anticoagulation. Vital signs at presentation were stable. A CT scan of abdomen/pelvis revealed a large amount of fluid in the upper abdomen with high attenuation material adjacent to the greater curvature of the stomach concerning for hemoperitoneum. Diagnostic laparoscopy revealed a significant amount of blood along the upper abdominal viscera. The procedure was converted to an upper midline laparotomy after identifying a necrotic, extremely friable 7 x 6 x 3 cm pedunculated mass with active hemorrhage on the posterior aspect of the greater curvature. A wedge resection was performed to remove the mass with grossly negative margins. An intraoperative frozen section revealed a stromal tumor with spindle cells. Final pathology revealed a pT3N0M0 stromal tumor with histologic spindle cells and a high mitotic rate (24/5 mm^2^) consistent with a high-grade GIST. Given tumor rupture at presentation, the patient was started on imatinib therapy for a minimum duration of three years. GISTs are often asymptomatic or cause mild abdominal pain or GI bleeding. Rarely, an exophytic GIST may rupture leading to intraperitoneal bleeding. Surgical resection with negative margins is the mainstay of treatment although patients presenting with tumor rupture are at higher risk of dissemination and recurrence.

## Introduction

Gastrointestinal stromal tumors (GISTs) comprise the largest subgroup of mesenchymal neoplasms [1]. The majority of these tumors are found in the stomach and small intestine although they can be found all throughout the GI tract. These tumors occur most commonly in patients older than 50 years of age. Seemingly related to the interstitial cells of Cajal, the majority of these tumors express CD34 and CD117, also known as c-kit [2]. Histologically, there are three subtypes of GISTs: the most common type is spindle cell, comprising up to 70%, followed by epithelioid and mixed type making up the remaining 30% [1]. Many GISTs are asymptomatic and identified incidentally. When symptoms occur, they commonly range from nonspecific nausea, bloating, and abdominal pain to gastrointestinal bleeding and rarely, intra-peritoneal hemorrhage [3,4].

In this case report, we present a rare occurrence in which an undiagnosed GIST presented with tumor rupture and spontaneous hemoperitoneum.

## Case presentation

**Figure 1:**
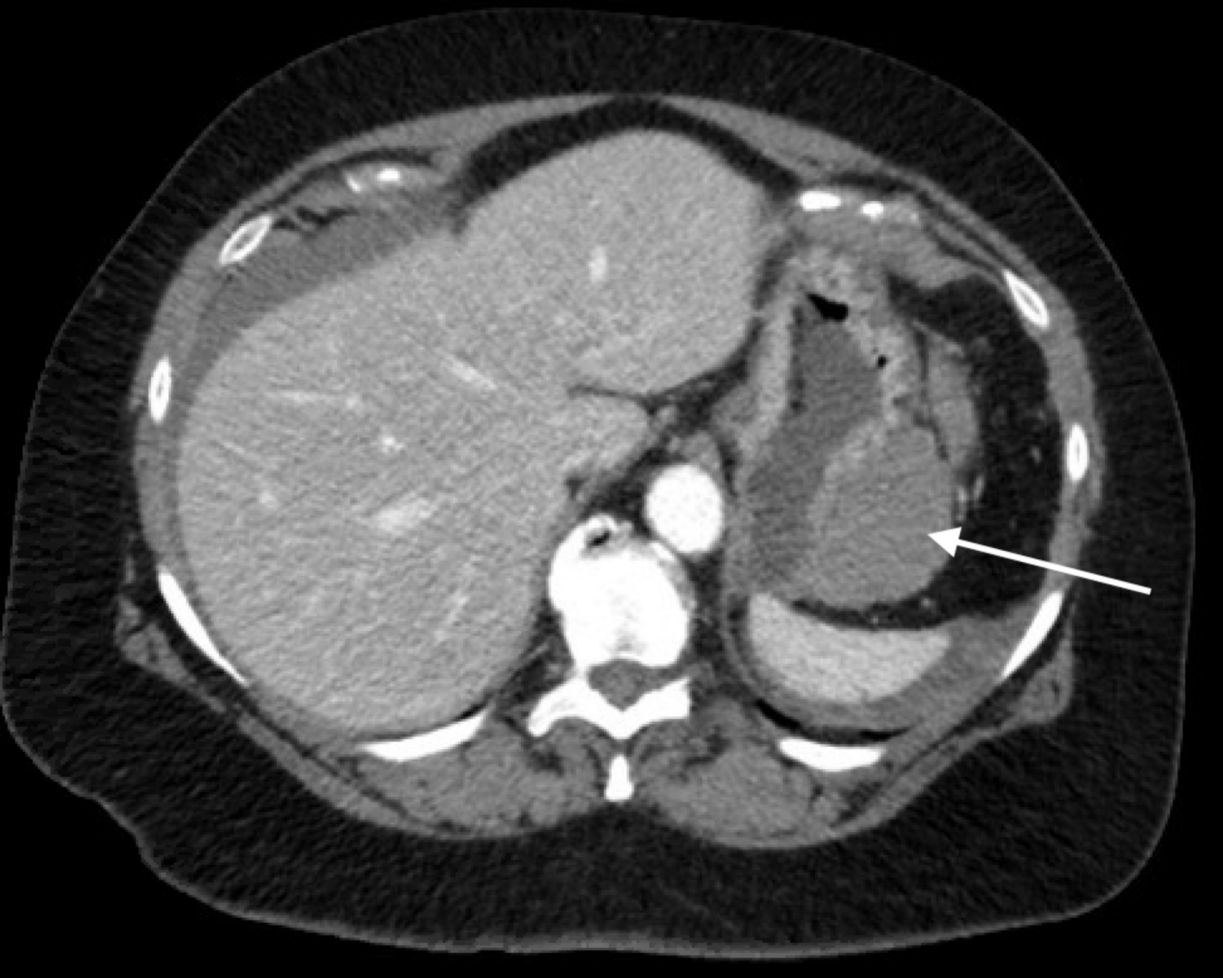
Axial view of the CT scan showing hemoperitoneum around the upper abdominal viscera and adjacent to the stomach (arrow)

**Figure 2:**
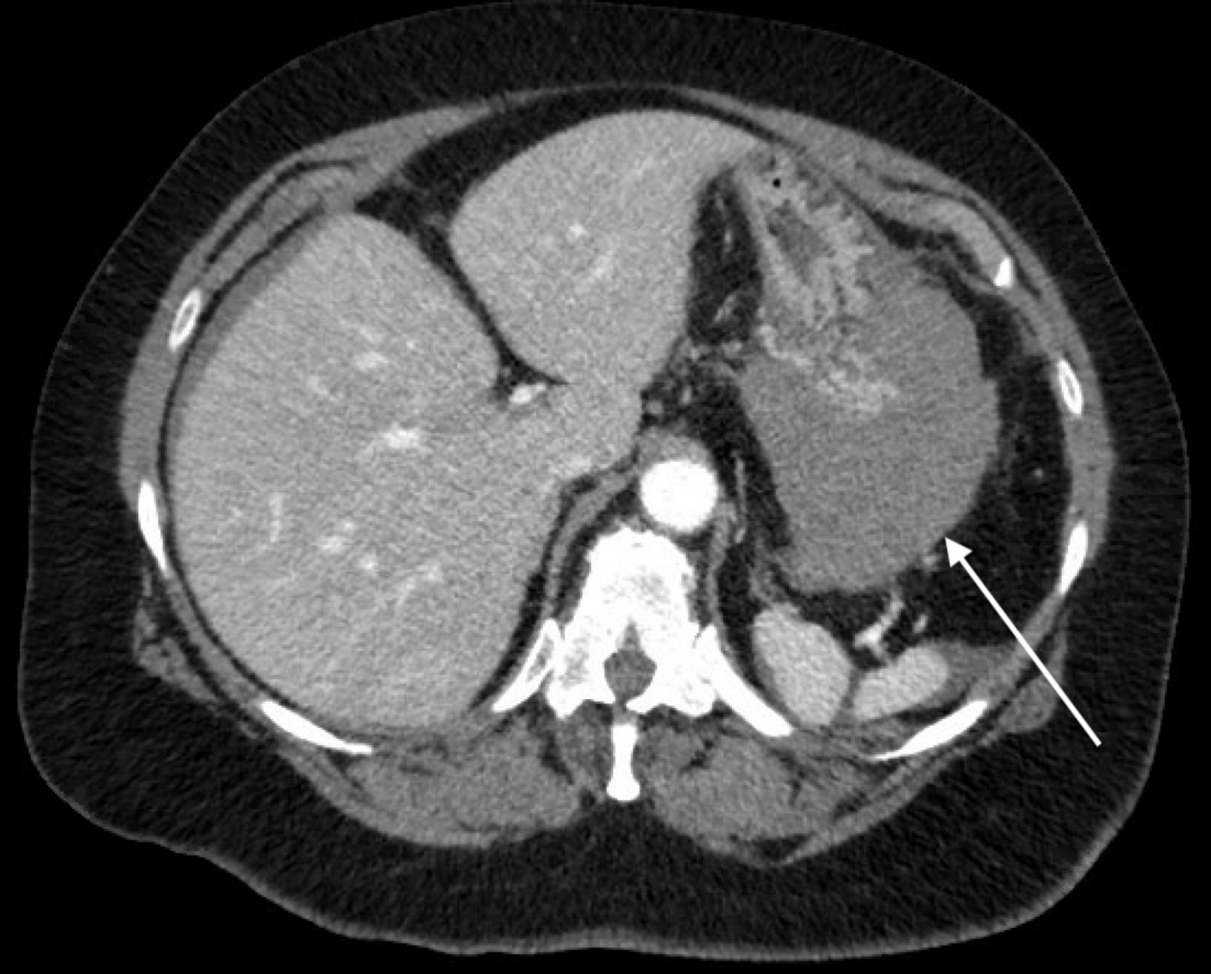
Axial CT image further distal showing high attenuation material adjacent to greater curvature of the stomach (arrow)

The patient was a 63-year-old female with a past medical history of hypertension and ulcerative colitis who presented to the emergency department with worsening epigastric pain. The pain started four days prior with one episode of non-bloody emesis, nausea, and one episode of dark stool. She denied previous history of abdominal surgery or trauma. She was not on any anticoagulant. Vital signs were within normal limits. On examination, the patient was diffusely tender to palpation, especially in the epigastrium. Initial lab work was unremarkable except for a mild leukocytosis of 12.3 k/uL and hemoglobin of 11 g/dL. A CT scan of abdomen/pelvis with intravenous contrast showed a large amount of blood in the upper abdomen (Figure 1) with high attenuation material adjacent to the greater curvature of the stomach (Figure 2) and around the liver consistent with hemoperitoneum, although a mass could not be excluded.

**Figure 3:**
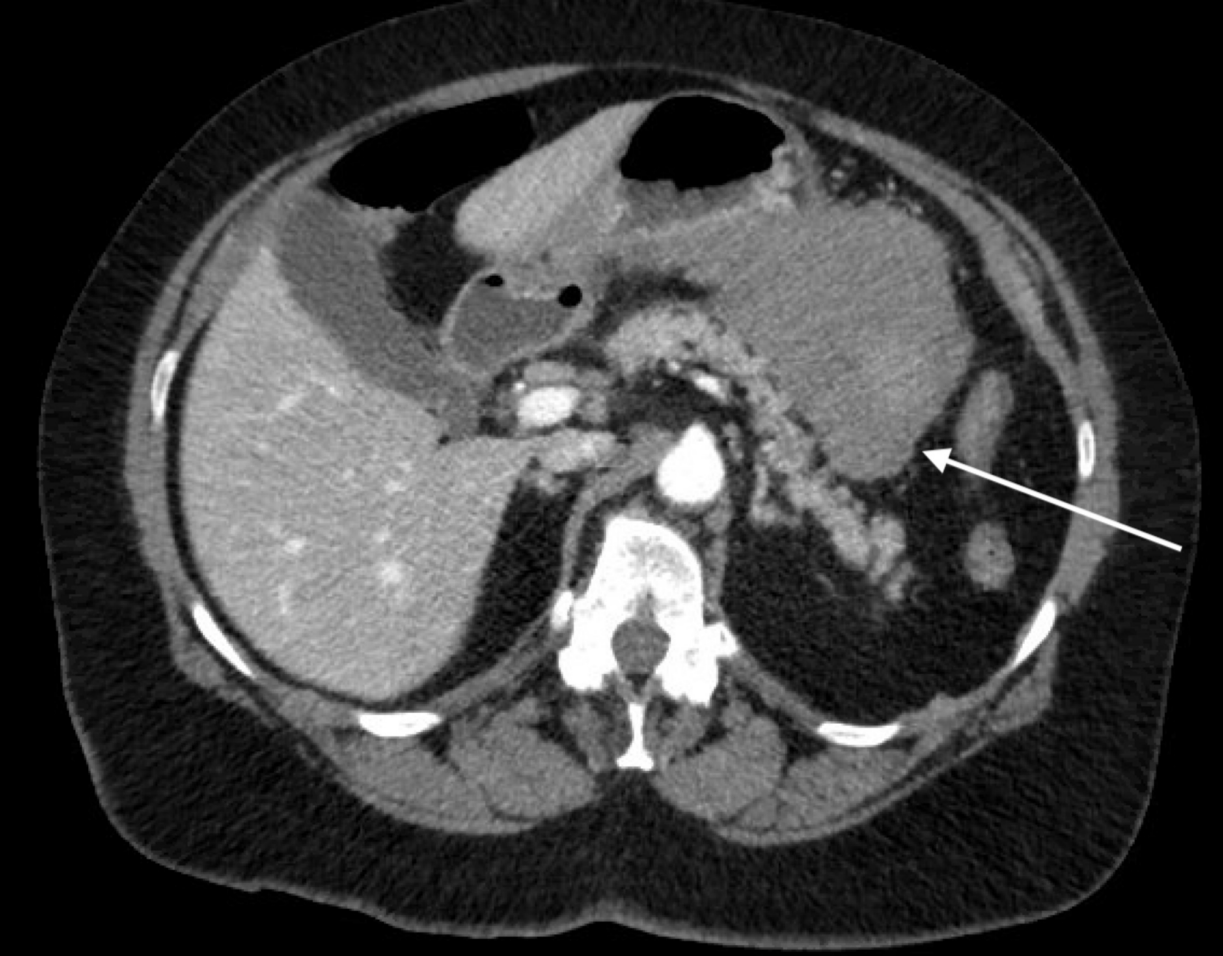
Axial CT scan showing a large amount of blood (arrow) in the lesser sac

Given the presence of hemoperitoneum and diffuse abdominal pain, the decision was made to undergo urgent diagnostic laparoscopy. Upon entering the abdomen, a large amount of blood was encountered around the upper abdominal viscera. The gastrocolic ligament was taken down to reveal an additional 800 mL of blood within the lesser sac as noted on preoperative imaging (Figure 3). An adherent clot was discovered on the posterior aspect of the stomach with a small amount of active extravasation. At this point, the procedure was converted to an open approach via an upper midline incision to adequately expose and control the source of bleeding. The stomach was retracted cephalad to expose the posterior body where a necrotic, friable mass was found along the superior aspect of the greater curvature. A partial gastrectomy was performed with a linear cutting stapler to wedge out the mass with grossly negative margins; the mass was found to be 7 x 6 x 3 cm with areas of necrosis and hemorrhage. An intraoperative upper endoscopy was performed to inspect the gastric and duodenal mucosa that appeared grossly normal. Intraoperative frozen section was positive for a stromal tumor with spindle cells. Final pathology of the mass demonstrated a high-grade gastrointestinal stromal cell tumor with spindle cells with positive KIT and Discovered on GIST-1 (DOG1) mutations. Postoperatively, she was admitted for observation and discharged without incident on postoperative day 3. The patient was referred to oncology and started on imatinib with planned duration of therapy of at least three years given tumor rupture at presentation.

## Discussion

GISTs are neoplasms of mesenchymal origin that appear to be related to the interstitial cells of Cajal [1]. Sixty percent of GIST tumors arise in the stomach followed by the small intestine and colon while rarely arising from the omentum or mesentery [1]. There are three histological subtypes of GIST although 70% of tumors are spindle cell followed by epithelioid and a mixed variant. These tumors appear to be more common in men. They may occur at any age although the majority of tumors are identified after the age of 50 years [2].

GISTs may be differentiated from other mesenchymal tumors based on gene expression. Over 95% of GISTs express CD117, or c-kit, which is a tyrosine kinase growth factor receptor distinguishing this tumor from leiomyomas, leiomyosarcomas, or schwannomas [2]. The expression of c-kit allows the use of a tyrosine kinase inhibitor like imatinib to be used in the neoadjuvant and adjuvant setting to increase disease-free survival. Rarely, GISTs may not express c-kit and instead contain a platelet-derived growth factor receptor alpha (PDGFRA) mutation or a subset referred to as wild type GIST without either mutation thus rendering imatinib therapy less effective [1]. The majority of GISTs occur sporadically, but they can be associated with hereditary syndromes such as neurofibromatosis type I [4].

The most common presentation of GISTs is nonspecific; vague abdominal pain, mild gastrointestinal bleeding, and laboratory evidence of anemia are the most common presenting symptoms. Occasionally, the patient can develop symptoms of bloating or obstruction due to mass effect. Rarely, GISTs can present with massive gastrointestinal bleeding, perforated viscus, or spontaneous hemoperitoneum [5,6].

Surgical resection is the mainstay therapy for managing GISTs with the goal to obtain a microscopically (R0) negative margin. Lymphadenectomy is not required due to the fact that these tumors rarely metastasize to lymph nodes. Tumor rupture, as in our patient’s case, increases the risk of recurrence and can lead to intraperitoneal spread. Due to the expression of c-kit in the majority of these tumors, tyrosine kinase inhibitors such as imatinib are effective in both the neoadjuvant and adjuvant settings in increasing progression-free and overall survival in advanced tumors [7].

Our patient presented with nausea, vomiting, and acute onset epigastric abdominal pain with CT evidence of hemoperitoneum. She had no prior abdominal surgical history and no free air was identified on imaging to suggest a perforated ulcer. The preoperative CT scan did not clearly identify the mass due to the surrounding intraperitoneal hemorrhage. Once the source of the bleeding was found to be secondary to a friable gastric mass at the time of operation, a GIST was suspected. As previously mentioned, surgical resection is the mainstay of therapy for a GIST and the mass was resected with grossly negative margins. Postoperatively, the patient was referred to medical oncology and imatinib therapy was initiated with a planned duration of at least three years given the high mitotic rate (24/5 mm^2^) and presence of tumor rupture. She will undergo CT scanning of the chest, abdomen, and pelvis every three to six months for three to five years followed by annually thereafter.

## Conclusions

In conclusion, a ruptured GIST should be in the differential when a patient presents with spontaneous hemoperitoneum without an obvious underlying cause. In patients with resectable disease, the mainstay of treatment is palliative surgical resection along with the addition of tyrosine kinase inhibitors for advanced tumors. Unfortunately, patients with a GIST who present in this manner have shorter overall survival and are at an increased risk of recurrence despite surgical and medical therapy.
